# Anesthesiologists’ Perspective on the Use of Artificial Intelligence in Ultrasound-Guided Regional Anaesthesia in Terms of Medical Ethics and Medical Education: A Survey Study

**DOI:** 10.5152/eurasianjmed.2023.22254

**Published:** 2023-06-01

**Authors:** Yasemin Koçer Tulgar, Serkan Tulgar, Selin Güven Köse, Halil Cihan Köse, Gülten Çevik Nasırlıer, Meltem Doğan, David Terence Thomas

**Affiliations:** 1 Department of Medical History and Ethics, Kocaeli University Faculty of Medicine, Kocaeli, Turkey; 2 Department of Medical History and Ethics, Samsun University Faculty of Medicine, Samsun, Turkey; 3 Department of Anaesthesiology and Reanimation, Samsun University Faculty of Medicine, Samsun Training and Research Hospital, Samsun, Turkey; 4 Department of Pain Medicine, Health Science University, Derince Training and Research Hospital, Kocaeli, Turkey; 5 Department of First and Emergency Aid Program, İstanbul Şişli Vocational School, İstanbul, Turkey; 6 Department of Medical Education, Maltepe University Faculty of Medicine, İstanbul, Turkey; 7 Department of Pediatric Surgery, Maltepe University Faculty of Medicine, İstanbul, Turkey

**Keywords:** Medical education, medical ethics, anesthesiology, ultrasonography

## Abstract

**Objective:**

Controversy exists around the world as experts disagree on what artificial intelligence will imply for humanity in the future. Medical experts are starting to share perspectives on artificial intelligence with ethical and legal concerns appearing to prevail. The purpose of this study was to determine how anesthesiology and reanimation specialists in Turkey perceive the use of artificial intelligence in ultrasound-guided regional anesthetic applications in terms of medical ethics and education, as well as their perspectives on potential ethical issues.

**Materials and Methods:**

This descriptive and cross-sectional survey was conducted across Turkey between July 1 and August 31. Data were collected through an online questionnaire distributed by national associations and social media platforms. The questionnaire included questions about the descriptive features of the participants and the possible ethical problems that may be encountered in the use of artificial intelligence in regional anesthesia and 20 statements that were requested to be evaluated.

**Results:**

The average age of the 285 anesthesiologists who took part in the study was 42.00 ± 7.51, 144 of them were male, the average years spent in the field was 10.95 ± 7.15 years, 59.3% were involved in resident training, and 74.7% habitually used ultrasound guidance regional anesthetic applications. Of the participants, 80% thought artificial intelligence would benefit patients, 86.7% thought it would benefit resident training, 81.4% thought it would benefit post-graduate medical education, and 80.7% thought it would decrease complications in practice. There will be no ethical issues if sonographic data are captured anonymously, according to 78.25%, while 67% are concerned about who will be held accountable for inaccuracies.

**Conclusion:**

The majority of anesthetists believe that using artificial intelligence in regional anesthetic applications will decrease complications. Although ethical concerns about privacy and data governance are low, participants do have ethical worries about “accountability for errors.”

**Main Points**

- The majority of anesthetists believe that using artificial intelligence (AI) in regional anesthetic applications will decrease complications.
- This survey found that the majority of anesthetists in Turkey believe that the use of AI in regional anesthetic applications will be advantageous for both resident training and post-graduate medical training.
- Using AI in regional anesthesia applications under ultrasound supervision would benefit the patient, resident training, and postgraduate continuing medical education, as well as lessen problems in both training stages.

## Introduction

Artificial intelligence (AI) or machine learning systems are computer systems that mimic human cognitive functions to execute tasks such as learning, problem-solving, and discrimination autonomously.^1^ In recent years, AI has found its place in nearly all fields where technology is utilized, facilitating and alleviating challenges in human existence.^2^ The most important advantages of AI technology are its ability to recognize structures and objects with high sensitivity and specificity, to provide fast reports attributed to programmed algorithms, and to achieve high consistency in results.^2^ Initially, AI was employed for goals such as facilitating diagnosis from radiological images. Furthermore, it is currently employed in nearly all sub-branches of medicine, including diagnosis, treatment, drug development technology, and even improving the doctor–patient interaction.^3-7^ Artificial intelligence is also being utilized to improve medical education.^8^

Artificial intelligence has been used in anesthesia for the estimation of adverse effects and mortality and the estimation of postoperative critical care requirements.^9-11^ The use of AI in ultrasound-guided (USG) regional anesthesia (RA) has been addressed in a variety of ways, including increasing the safety of RA, decreasing the complication rate, as well as being used in education, particularly as an innovation to assist in the identification of sono-anatomy for USG RA where it has been reported to be highly popular and motivating.^12-17^

In RA practice, AI is utilized in USG-guided RA applications. Artificial intelligence can identify all vital sono-anatomical structures, display the target, and help the practitioner to advance the needle tip to the correct target.^13,16^ Every innovation is accompanied by unprecedented challenges. It appears that AI, as is the case in RA education, will break new ground in the world of education, with innovation as well as new concerns. Although there is growing consensus about the benefits and advantages of AI, ethical and legal concerns still remain. Prospective practitioners are at best to anticipate these benefits and potential problems.

The purpose of this survey is to ascertain the opinions of Turkish anesthesiology and reanimation specialists on the ethical implications of the use of AI in USG RA.

## Materials and Methods

The study was approved by the local ethics committee. Surveys were sent to anesthesiology and reanimation specialists working in Turkey, between July 1 and August 31, 2022. 

The study's questionnaire was originally written and applied in Turkish. A translation into English made by our English-native co-author (DTT) is included as a supplement (Table 1). All participants consented to participation, processing of data processing, and inclusion of their data in medical research, at the beginning of the survey. Those who did not consent were unable to access the questionnaire. There were a total of 5 descriptive items: age, gender, years of experience in the profession, participation in resident training, and routine use of USG in RA applications in the operating room.

All participants were given information about AI and its use in RA practice. Participants were asked to answer a total of 7 main questions with a total of 20 statements, all with regards to the use of AI in RA:

### Medical Ethics – Beneficence

In the first question (Q1), participants were asked to evaluate beneficence for patients in light of medical ethics principles.

### Medical Ethics – Maleficence

In Q3/S4 and Q4/S3, participants were asked to evaluate the AI use in light of the medical ethics concept of “Maleficence.” 

### Medical Education and Ethics – Beneficence

In Q3/S1-3 (resident training) and Q4-S1 (continuing medical education after graduation), participants were asked to evaluate the use of AI in terms of the “beneficence” principle of medical ethics and education.

### Medical Education – Equal Opportunity

In Q2 (resident training) and Q4/S2 (continuing medical education after graduation), participants were asked to evaluate the use of AI in terms of equal opportunity in education.

### Ethical Concerns – Accountability for Errors

In Q5/S1-4 and Q6/S1-4, participants were asked to evaluate the use of AI in terms of “accountability for errors” in residency training and postgraduate continuing medical education, respectively.

### Ethical Concerns – Data Governance and Privacy

In Q7/S1-4, participants were asked to evaluate AI in terms of “data governance and privacy.”

On a 3-point Likert scale, participants evaluated a total of 20 judgments. In addition, participants were given the chance to share any ethical issues that were not addressed in the questionnaire in a final open-ended question. Table 1 provides an example of the questionnaire's English translation.

The questionnaire, which was produced as a Google Form, was distributed on social media such as WhatsApp, Twitter, and Facebook to anesthesiologist groups and also via individual e-mails. Considering the size of the population (approximately 6500 specialists), a minimum sample size of 262 participants was calculated using a 90% confidence level and a 5% margin of error. Due to the possibility of data loss, a minimum of 300 participants were intended.

### Statistical Analysis

Statistical Package for the Social Sciences 16 was used for data analysis (SPSS Inc., Chicago, IL, USA). Descriptive data were given as mean and standard deviation, and survey responses were given as frequency and percentage. *T*-test was used in the analysis of descriptive data. Categorical data were presented as counts and percentages and compared using Chi-square test or Fisher’s exact test as appropriate, with post-hoc Bonferroni adjustments to determine where the difference between groups originated. Statistical significance was accepted as *P* < .05. 

## Results

A total of 305 participants completed our survey within the specified date range. Although the introduction section of our survey clearly stated that only specialists were invited to participate, we determined that 20 participants were residents continuing their training and thus they were excluded from the study. The female/male ratio of the participants in our study was 141/144. The mean age of participants was 42 ± 7.51 years. The mean length since completing training was 10.95 ± 7.15 years (range 0-30 years).

Of the participants, 59.3% took an active role in resident training, and 74.7% stated that they routinely used USG in RA practices. Descriptive data are presented in Table 2.

Of the participants, 80% agreed (Q1) that the use of AI in RA would be beneficial for the patient. The percentage of participants that stated AI would be beneficial in resident and postgraduate education (Q3-S1, Q3-S2) was 86.7% and 81.4%, respectively. Of the participants, 65.26% and 69.47% stated that the use of AI would lead to equal opportunity in education for both residents and in postgraduate education programs, respectively. Participants agreed that AI would lead to a decrease in complications for residents in training (Q3-S4, 81%) and for specialists too (Q4-S3, 80.7%).

There was no consensus over who would be accountable for a potential complication in AI-assisted practices. Of the respondents, 51% agreed that both the trainer/practitioner and AI should be held accountable for issues arising from the training of residents and the practice of specialist physicians. In all instances, nearly 67% of respondents agreed that the question of who will be accountable for complications if they occur would be problematic. 

Only 9.47% of participants believed that it would be an invasion of privacy for AI to save the sonographic data of patients in their memory, while 78.25% thought that it would not be an issue to record this data anonymously. Of the participants, 67.7% stated that patients should be able to revoke their agreement for the use of their data at any time. 

**Figure 1.**
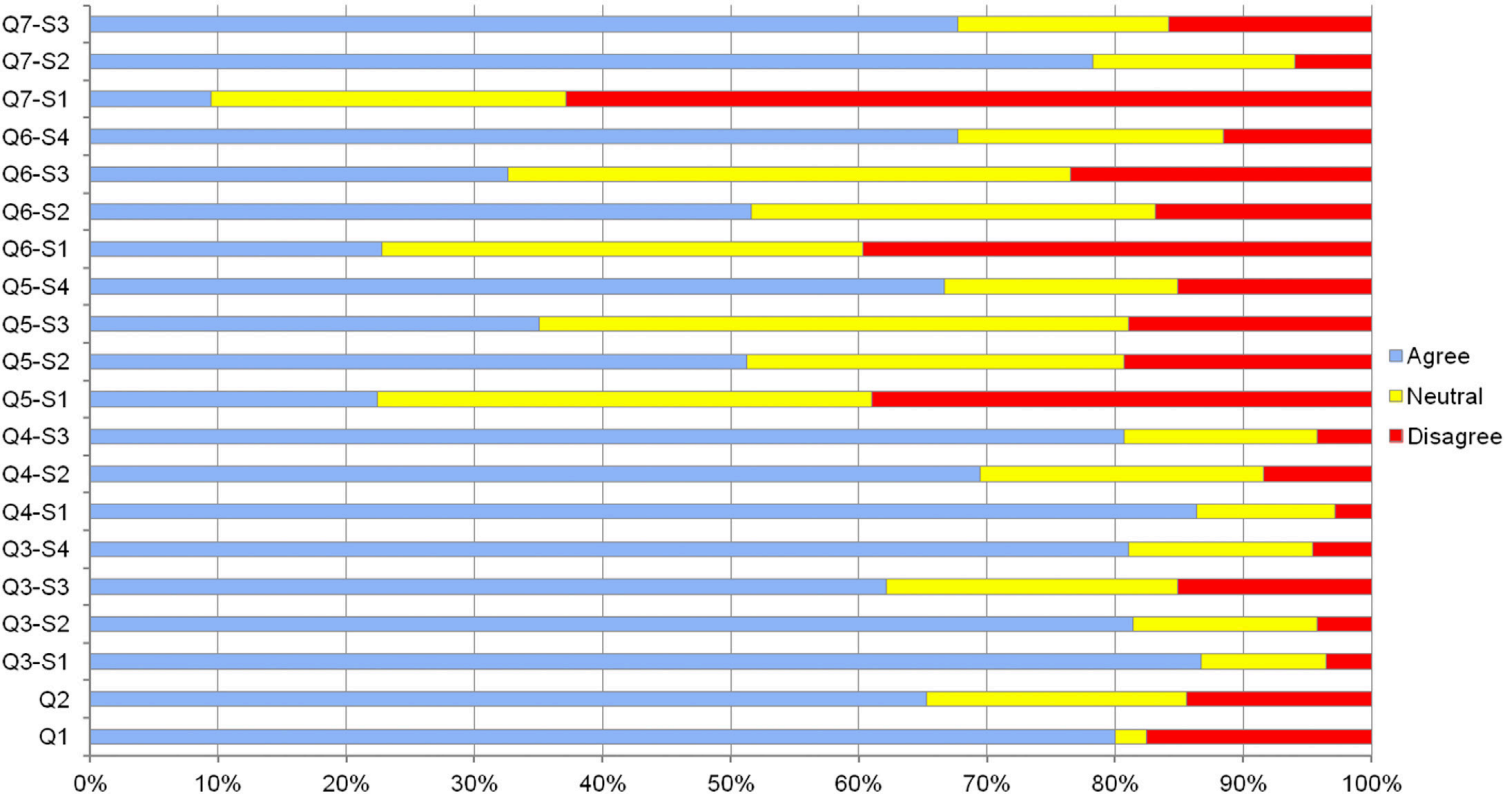
Distribution (percentage) of the answers given by the participants per question.

Participants were also asked for their additional opinions if any. We observed that reinforcing statements about the judgments presented in the survey statements were generally repeated. Participant feedback stating that sonographic data are not recorded in current AI applications was received. This was not in contraction to the root of our question. Figure 1 depicts the percentage of responses to all questions and statements.

When responses to survey questions were analyzed according to descriptive characteristics of participants such as age, gender, time spent in the profession, routine USG use, and active participation in assistant training, statistical significance was determined in only 3 judgments (*P* > .05). While 68.8% of those who actively participated in the training of residents agreed that the use of AI in RA would improve the relationship between the trainer and trainee, only 52.6% of those who did not participate in resident training agreed with this statement (*P* = .02). In Q6-S1, the statement that the responsibility will be solely on the practitioner in case of a complication during the use of AI was presented to the participants. Of the participants, 26.8% who routinely use USG in RA applications agreed with this statement, while 11.1% of the participants who do not use USG agreed. This difference was found to be statistically significant (*P* = .02) (Table 3).

## Discussion

Our study found that the majority of participants believed that using AI in RA applications under ultrasound supervision would benefit the patient, resident training, and postgraduate continuing medical education, as well as lessen problems in both training stages. Furthermore, it was established that responsibility sharing would be a concern in the event of difficulties or complications that may emerge in AI-assisted RA applications during resident training and thereafter (about 70%). There was consensus that it was not against privacy to record data anonymously and to keep it in memory (75%). In addition, it was determined that the rate of agreement in the idea that AI programs keep patient data in their memory is contrary to privacy is quite low (10%).

The use of AI in medicine has increased in popularity, particularly in the previous decade.^2^ The USG applications in RA have grown in popularity in recent years. We asked participants in our study to rate the utility of AI help in RA training during resident training and postgraduate continuing education. In the near future, it appears that the use of AI in medical education will lead to a number of improvements similar to those in all areas of medicine. However, as the data fog grows with advances in technology, it becomes increasingly important to update and improve educational standards.^18^ The long journey ahead requires the collaboration of medical science and data scientists. An evaluation paper on the application of AI in medicine predicts that it will have significant benefits in both decision-making in patient diagnosis and treatment, as well as in medical education.^19^ Participants in our study believed that AI guidance in RA applications would be beneficial both in resident training and in continuing medical education after graduation. If we think of AI as a product in RA practice, we can see that the product's target audience is willing to use it and believes in its educational benefits.

Despite being used interchangeably in the literature, AI and machine learning are not synonymous.^20^ Artificial intelligence uses machine learning algorithms such as reinforcement learning algorithms and deep learning neural networks are used. Machine learning, on the other hand, allows a computer system to generate predictions or make decisions based on past data without being explicitly programmed. Machine learning is based on an algorithm that learns on its own through the use of previous data. Artificial intelligence is a technology that allows machines to mimic human behavior. The goal of AI is to create a clever computer system that can solve complicated issues like humans. The purpose of machine learning is to enable machines to learn from data in order to provide correct output. Although we used the term AI in our work, the system currently being used in research is machine learning or deep machine learning systems.

To create a categorized summary, we found that a majority of anesthetists in Turkey (>80%) believe that employing AI in RA applications will benefit the patient, which is one of the core principles (*Beneficence*) of medical ethics.^21^ We also addressed another major principle, nonmaleficence, in an indirect manner. More than 80% of our participants thought that AI assistance would help to lessen problems in RA training and practice. We believe that AI can help practitioners not only take action for the benefit of the patient but also avert potential harm.

When we evaluate the results of our survey in terms of medical education, we discovered that the majority of participants (>80%) believed that incorporating AI into RA training will benefit trainers/trainees and practitioners. Again, we discovered that the vast majority (about 70%) believed AI will contribute to equal opportunity in medical education. What about equitableness? It should not be forgotten that medical education also has ethics and that the ethics of medical education cannot be treated separately from medical ethics.^22,23^ It is necessary to conduct research to determine AI's role in RA teaching and its possible benefits. If it does indeed promote equal opportunity, its dissemination can help to resolve the dilemma of injustice in medical education, or alternatively it may pose further questions.^24,25^

According to an article, the primary ethical challenges that arise from using AI in surgery include human agency, accountability for errors, technical robustness, privacy and data governance, openness, diversity, non-discrimination, and justice.^26^ In our study, we assessed anesthetists' perspectives on the accountability for errors item in question root 5 (applications in resident training) and question root 6 (applications performed by experts). In question root 7, we addressed privacy and data governance. Other ethical considerations can be addressed in future research with larger Delphi investigations.

Approximately two-thirds of the participants believed that the issue of “accountability for errors” in the employment of AI in RA training for residents would constitute an ethical concern. Half of the participants definitely believed that both AI and the trainer should be held accountable. It was found that the employment of AI in RA, like in other fields of medicine, will raise an ethical quandary in terms of “accountability for errors.”^27^ Although there have yet to be any legal ramifications (since there are no laws in this area), in the near future, if the systems utilized are updated from machine learning to sophisticated AI, the subject of who will be held accountable may spark heated debate.

Privacy and data governance are 2 other potential ethical issues. Systems developed for RA guidance, as is well known, are systems with recognition models that mark adjacent anatomical tissues and target tissue using a machine learning (or deep machine learning)-based processor that processes historical data (obtained in accordance with ethical guidelines) using certain algorithms. These programs are not involved in decision-making. They do not constantly collect data to better themselves as human intelligence does. However, as previously stated, AI is much more than machine learning. It solves problems by making decisions on its own and drawing new conclusions by analyzing the facts presented to it over time. When the process reaches this point, privacy and data governance may become of more ethical importance.

However, when we look at current practices, we observe that sonographic data from patients are neither regularly used in repetition nor stored in machine learning. However, the procedure will significantly differ from the continuous learning and guiding features of AI. Although there is currently no example, AI will analyze fresh data in each application, compare it to its previous “experiences,” and achieve goals beyond what was envisaged. We asked participants in the seventh question of our questionnaire to evaluate – ethically, a circumstance that has not yet been experienced but that we may have to confront in the near future: storage of sonographic images in the memory of the AI. From an ethical standpoint, a relatively tiny number of the participants (10%) believed this would be an “infringement of privacy.” Approximately 80% felt that recording sonographic data anonymously would not pose an ethical issue. When we conducted a literature review, we discovered studies that imply the advantages of AI's access to very large databases, as well as those that pose ethical concerns.^26,28,29^ We believe that there is currently no cause for concern regarding AI in RA.

Is there a lack of standardization and norms for the use of AI in medicine, and what ethical problems must be addressed? As a future insight, we can argue that each branch of medicine will need to put forward their own worries regarding the use of AI in their field.^26^ In the near future, professional associations, physicians, ethicists, law-makers, AI developers, and patient rights advocates will need to address problems and find consensus solutions regarding the medicolegal and ethical elements of AI.^30^ Product developers should be more explicit about how data security is supplied in AI, and practitioners' concerns should be addressed. Of course, potential users' ethical concerns should also be resolved ahead of time by completing studies like ours. Given that AI is progressing from analyzing radiological imaging such as x-rays, mammography, and tomography to assessing and recognizing races, we must realize that these systems must be addressed from several perspectives (including hazards such as discrimination) and that we are only at the tip of the iceberg.^31^

Our study has some limitations. First, although we calculated the sample size, the study might have been strengthened by expanding the number of participants using a nationwide random sampling that takes into account variables such as age, gender, employer, and length of employment. We built the ethical concerns section of our study on the 3 primary ethical concerns that we anticipated. We could have, however, through a Delphi study, determined the major titles and their quantities in advance and thus conducted a more thorough examination.

This survey found that the majority of anesthetists in Turkey believe that the use of AI in RA applications will be advantageous for both resident training and post-graduate medical training and will reduce complications. We also determined that privacy and data governance pose few ethical concerns. The majority of respondents said that these activities would raise ethical problems around “error accountability.”

## Figures and Tables

**Table 1.** Question Roots and Statements of Survey

	Agree	Neutral	Disagree
Q1. Use of artificial intelligence in regional anesthesia applications will benefit the patient.			
Q2. It will provide equal opportunity among residents studying in different institutions.			
Q3. The use of artificial intelligence in resident training in regional anesthesia applications;			
S1. It will be useful for residents			
S2. It will be useful for educators			
S3. It will positively affect trainer–trainee relationship			
S4. It may reduce the complications that could occur during the learning period			
Q4. When the introduction of artificial intelligence into regional anesthesia practice is evaluated in terms of continuing medical education after graduation;			
S1. It will be beneficial for the professional development of anesthesia and reanimation specialists.			
S2. It will be beneficial in terms of equal opportunity in education.			
S3. It may reduce the complications that could occur in regional anesthesia applications.			
Q5. In case of “a complication due to misidentification and orientation of the program” in artificial intelligence-supported applications in resident training;			
S1. The responsibility should be solely on the trainer.			
S2. Responsibility should not be only on the trainer, artificial intelligence can also be held responsible.			
S3. Responsibility should lie with the manufacturers or programmers of artificial intelligence.			
S4. Complications during training will create a problem about who will be responsible.			
Q6. In the case of “a complication due to misidentification and guidance of the program” in artificial intelligence-supported applications performed by anesthesiologists;			
S1. The responsibility should be solely on the trainer.			
S2. Responsibility should not be only on the trainer, artificial intelligence can also be held responsible.			
S3. Responsibility should lie with the manufacturers or programmers of artificial intelligence.			
S4. Complications during training will create a problem about who will be responsible.			
Q7. Artificial intelligence programs are currently guiding regional anesthesia by recognizing tissues through sono-anatomical images obtained with “ethics committee approval” and “patient consent.” However, it is not plausible to obtain such approval/consent continuously as data from sequential applications lead to more data with increased sensitivity. Storage of sonographic images in the memory of artificial intelligence programs that processes these images in order to guide the practitioner better;			
S1. It is against privacy principles.			
S2. Since patients are recorded anonymously, it cannot be considered as a violation of privacy.			
S3. Patients should be able to withdraw their consent at any time.			
8. If you believe there are other ethical problems related to the use of artificial intelligence in regional anesthesia, please share your thoughts with us.			

**Table 2.** Demographic Characteristics of Participants

Variable	Result
Age (years)	42 ± 7.51
Sex (n, %)	
Female	141 (49.5%)
Male	144 (50.5%)
Active role as trainer? (n, %)	
Yes	169 (59.3%)
No	116 (40.7%)
Duration of experience as an anesthesiology specialist	
<10 years	113 (39.6%)
>10 years	172 (60.4%)
Using ultrasound in regional anesthesia: (n, %)	
Yes	213 (74.7%)
No	72 (25.3%)

**Table 3.** Assessment of Descriptive Items Regarding Questions/Statements

Question No		Agree	Neutral	Disagree	*P*
		n (%)	n (%)	n (%)	
Active role in residency education					
Q3-S3	Yes	116 (68.6%)*	33 (19.5%)	20 (11.8%)	.021
	No	61 (52.6%)	32 (27.6%)	23 (19.8%)	
Using US in regional anesthesia					
Q6-S1	Yes	57 (26.8%)	75 (35.2%)	81 (38%)	.023
	No	8 (11.1%)*	32 (44.4%)	32 (44.4%)	

No, number; Q, question; S, section; US, ultrasound.

Values are presented as number (%).

*Indicates the group leading to statistically significant difference.
